# Can Zipf's law be adapted to normalize microarrays?

**DOI:** 10.1186/1471-2105-6-37

**Published:** 2005-02-23

**Authors:** Tim Lu, Christine M Costello, Peter JP Croucher, Robert Häsler, Günther Deuschl, Stefan Schreiber

**Affiliations:** 1 Department of Medicine, Christian-Albrechts-University, Kiel, Germany; 2 Department of Neurology, University Hospital Schleswig Holstein, Kiel, Germany; 3 The Conway Institute for Biomolecular and Biomedical Research, University College Dublin, Ireland

## Abstract

**Background:**

Normalization is the process of removing non-biological sources of variation between array experiments. Recent investigations of data in gene expression databases for varying organisms and tissues have shown that the majority of expressed genes exhibit a power-law distribution with an exponent close to -1 (i.e. obey Zipf's law). Based on the observation that our single channel and two channel microarray data sets also followed a power-law distribution, we were motivated to develop a normalization method based on this law, and examine how it compares with existing published techniques. A computationally simple and intuitively appealing technique based on this observation is presented.

**Results:**

Using pairwise comparisons using MA plots (log ratio vs. log intensity), we compared this novel method to previously published normalization techniques, namely global normalization to the mean, the quantile method, and a variation on the loess normalization method designed specifically for boutique microarrays. Results indicated that, for single channel microarrays, the quantile method was superior with regard to eliminating intensity-dependent effects (banana curves), but Zipf's law normalization does minimize this effect by rotating the data distribution such that the maximal number of data points lie on the zero of the log ratio axis. For two channel boutique microarrays, the Zipf's law normalizations performed as well as, or better than existing techniques.

**Conclusion:**

Zipf's law normalization is a useful tool where the Quantile method cannot be applied, as is the case with microarrays containing functionally specific gene sets (boutique arrays).

## Background

DNA microarrays have become a widely used biotechnology for assessing expression levels of tens of thousands of genes simultaneously in a single experiment [1,2]. Whether microarrays are being used for global tissue profiling or for differential expression studies, data normalization is an essential preliminary step before statistical analysis methods can be applied. The purpose of all normalization techniques is to transform the data to eliminate sources of variability stemming from experimental conditions, leaving only biologically relevant differences in gene expression for subsequent analysis. Normalization can be divided into two stages, intra-array normalization and inter-array normalization. Intra-array normalization deals with variability within a single array caused by factors such as differences in print-tip characteristics, channel differences in two-dye systems, and spatial heterogeneity across the array surface [3-5] and should be carried out using accepted methods before inter-array normalization is applied. This paper assumes intra-array normalization has been performed and presents an inter-array normalization method for comparison of gene intensity levels between multiple microarrays to deal with variation caused by such factors as differences in RNA isolation efficiency, labeling efficiency, hybridization conditions, exposure times, and detection efficiencies.

It is now clear that simple inter-array normalization techniques, such as simple scaling to housekeeping genes or normalizing to a global mean, are not adequate for microarray data [6]. Housekeeping genes have been found to be more susceptible to modulation than previously thought [7]. Along with others [5], this paper underscores the potentially serious drawbacks of the global mean and other such methods. Recent literature has thus provided a plethora of more sophisticated normalization and analysis techniques as researchers struggle to cope with the task of microarray data analysis, some of which include maximum likelihood analysis [5], centralization [6], principal component analysis [8], analysis of variance [9] and Bayesian network analysis [10].

Analysis of publicly available large-scale SAGE gene expression data sets [11,12] and an intra-phyletic survey of genome wide Affymetrix microarray experiments [13] have indicated that the large majority of expressed genes exhibited power-law distributions, while some microarray expression data exhibit a more log-normal distribution [14]. Our normalization procedure was inspired by the observation that the intensities measured on our microarray system also followed a power law distribution and can therefore be described by a simple mathematical model. Zipf's law [15] is a power law function that states that the magnitude of an intensity measurement (*y*) is inversely proportional to the rank (*r*) of that data point in the data set,

*y*∝*r*^*c *^    (1)

where *c *is a coefficient close to -1. Our microarray data can be classified as a generalized form of Zipf's law because the coefficient (*c*) is not always close to -1 and, in fact, varies between individual microarrays, making simple linear normalization procedures, such as global normalization to the same mean, inappropriate. However, the normalization procedure proposed here demonstrates that by taking Zipf's law into account, it is possible to apply a simple intra-array normalization procedure such that all filters have the same coefficient *c *and proportionality.

We demonstrate the Zipf's law based normalization technique on microarray data sets representing both single channel and two channel technologies. In the single channel category, we produced two radio-labeled, nylon membrane based cDNA data sets, one commercial and one generated "in-house". Both systems contain a selection of genes chosen without regard to functional or pathway considerations, which make them especially appropriate for normalization using Zipf's law. These data sets were also normalized to a global mean (the mean of all microarrays) [16], and the quantile normalization method [17]. In addition we produced a two channel, fluorescently labeled, glass slide, oligo-based microarray data set generated 'in-house'. This microarray can be classified as a 'boutique' microarray because it consists of a selection of genes involved in apoptosis. This data set was normalized with a variant of the Zipf's law normalization method that uses a subset of the distribution as a proxy for normalizing the entire microarray. A comparison was then conducted against a variant of the loess normalization method that uses an *a priori *selection of 'housekeeping' genes as a proxy for normalization.

The finding that our microarray data distributions conform to a power law distribution agrees with predictions based on genome wide gene expression studies [11-13], however Hoyle, *et. al. *[14] observed that microarray distributions were log normally distributed with possible power law tails. To investigate this discrepancy, and to verify that our normalization technique could be useful in the normalization of data sets from other microarray systems, we also surveyed publicly available data sets from the NCBI Gene Expression Omnibus [18].

The two assumptions upon which the normalization method are based are the same as those used in other normalization methods [5,6], namely that in comparisons between similar tissues or cell lines under different experimental conditions i) most genes are not, or only moderately, regulated, and ii) approximately equal numbers of genes are up regulated as down regulated. Systems which conform to these two assumptions will be referred to as 'well-behaved' in this paper. While these assumptions probably hold for microarrays derived from a diverse sampling of genes, for example an EST library survey, they may not hold for microarrays containing genes specifically selected based on function or pathway (so called 'boutique' microarrays) as it is likely that most genes will be affected by the experimental treatments. One way to circumvent the restrictions resulting from these assumptions is to use a subset of data, or proxy, from the boutique array data set which fulfils the 'well-behaved' criteria. In developing a boutique microarray normalization technique, Wilson et. al. [4] have devised a method for selecting a subset of genes within a microarray data set that have low variation between arrays and are well representative of the spectrum of intensities measured on the microarray. They term this *a priori *selected subset 'housekeeping' genes, however it should not be confused with the *a posteriori *set of genes typically envisioned when the term is used. Another possible proxy that could meet the 'well-behaved' criteria are control spots which are included on the microarray during it's manufacture. We tested our normalization method on data from a two channel boutique microarray experiment using two types of control spots as proxies for normalization (Positive and negative internal controls, and housekeeping genes). The Zipf's law normalization methods were then compared with the variant of the loess method developed by Wilson et. al. [4] using housekeeping genes.

## Results

### Verifying Zipf's Law

**Figure 1:**
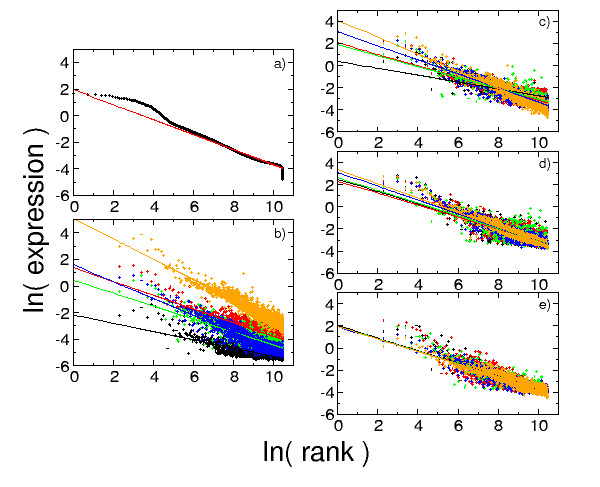
**Unigene microarray log plots. **Five human Unigene microarrays from the panel of thirty-one microarrays used in the sigmoidal colon experiments. Upper left to lower right: **a**. Log_e _median gene intensity vs. log_e _rank – conformity to Zipf's law is demonstrated by the linear regression line (in red) **b**. Five microarrays chosen to maximize pre-normalization variability, each plotted according to the gene ranks determined by their median gene intensity levels. **c**. The same five microarrays, normalized to a global mean, with regression lines. **d**. The same five microarrays, normalized with the quantile method, with regression lines. **e**. The same five microarrays, normalized taking Zipf's law into account, with regression lines. For plots b-d, a sub-sample of 10% of the data points are plotted for readability.

Before applying the described normalization method, the adherence of the reference curve (the median gene intensity data versus rank) to Zipf's law was verified. The most common method of verifying conformity to Zipf's law is a linear regression on the log_e_-log_e _transformed data set. Our regression showed a good fit, with a correlation coefficient of -0.98 and a slope of -0.56 for microarrays representing human colon (Figure 1a, Figure 6A, Table 1 set A), a correlation coefficient of -0.99 and a slope of -0.78 for rat brain microarrays (Figure 6B, Table 1 set B), and a correlation coefficient of -0.99 and a slope of -0.60 for the mouse apoptosis microarrays (Figure 6H, Table 1 set H). It should be noted that while the low ranking intensities may show a marked deviation from the regression line, this data typically accounts for a very small proportion of the total data and does not have a large affect on the regression curves.

### Normalization results – single channel microarrays

A comparison of the Zipf's law normalization method to the simple method of setting all arrays to a global mean (the mean of all microarrays) and to the quantile method was conducted on the single channel microarray data sets. Five human Unigene microarrays from the panel of thirty-two microarrays used in the sigmoidal colon experiments were selected to represent the greatest variability in pre-normalized data observed in the experiment (Figure 1b). Normalization to a global mean (Figure 1c) yielded data sets that displayed a higher variability in the coefficient *c *of the Zipf's power function (formula 1) than that observed after normalization by the Zipf's law method (Figure 1e) or the quantile method (Figure 1d). The Zipf's method showed the lowest variation in the Zipf's exponent and had the lowest spread of the data around the ln(rank) vs. ln(intensity) line. Results of an identical log_e _intensity versus log_e _rank plot comparison in Clontech rat microarrays showed little difference between the quantile and Zipf's methods [see Additional file 1]. However it should be mentioned that this method of data plotting provides one view of the data which is especially favorable to the Zipf's law normalization method. Next we examine the results of the MA-plots, a technique that is especially favorable to the quantile normalization method.

**Figure 2:**
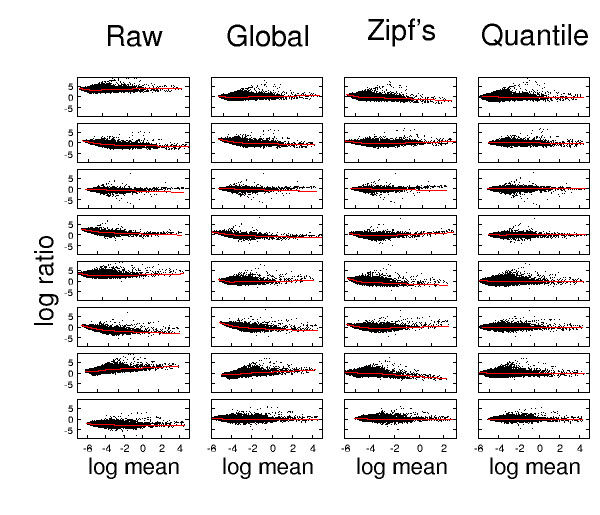
**Unigene microarray MA plots. **MA plots of Raw Unigene data compared to data normalized with the Global mean, Zipf's, and Quantile methods (columns). Each row of plots represents one pairwise comparison, only 8 of the possible 10 pairwise comparisons of the 5 microarrays used in figure 1are shown. Lowess curves are plotted in red.

**Figure 3:**
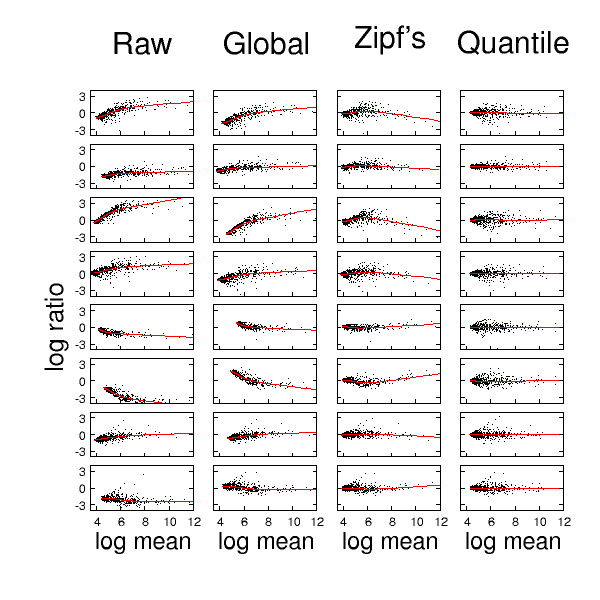
**Clontech microarray MA plots. **MA plots of Raw Clontech Rat data compared to data normalized with the Global mean, Zipf's, and Quantile methods (columns). Each row of plots represents one pairwise comparison, only 8 of the possible 10 pairwise comparisons of the 5 microarrays used in Additional file 1 are shown. Lowess curves are plotted in red.

In order to access the effectiveness of the normalization method, pairwise comparisons using MA-plots (sometimes called RI plots, or log ratio vs. log mean intensity plots) [19] were carried out on the raw data, and data normalized with the global mean method, quantile normalization and Zipf's law on both data set A & B (Figure 2 &3 respectively). With the raw data, the distribution of log-intensity ratios is not centered around zero which is as expected in an un-normalized data set. There is a noticeable intensity dependent effect, sometimes described as a 'banana' curve, which is characteristic of many microarray data sets. Normalization with the global mean method results in a shift of the center of the log-intensity ratio distribution closer to zero, one important criterion for well normalized data, however, especially in the low log mean range, the bulk of the data points still deviate appreciably from zero. The intensity dependent effect is evident, with the low intensity end of the loess fit curving away from the zero axis. The intensity dependent effect is removed using the quantile method. The log intensity ratios of the data distributions normalized using Zipf's law are well centered around zero, but the intensity dependent effect is still apparent. In this case however, the bulk of the data lies very close to zero on the log-ratio scale. [see Additional file 2] This is due to the fact that Zipf's law normalization not only shifts the data distribution on the log ratio scale, but also rotates the whole distribution in log-ratio log-intensity space.

The Kolmogorov-Smirnov test is often used to determine whether data distributions differ significantly and provides a test statistic that measures the proportion of overlap between distributions which ranges from 0 (in the case of identical distributions) to 1 (for non-overlapping distributions) [20]. Mean Kolmogorov-Smirnov values (Table 2a, b) showed the expected trend, with the high values for raw, unnormalized data decreasing when global median normalization was applied, decreasing again after Zipf's law normalization, and reaching zero for both data sets under quantile normalization. It should be noted that the Kolmogorov-Smirnov test statistic will always be zero after quantile normalization because this method forces the data distributions of all microarrays to be identical.

### Normalization Results – Two Channel Boutique Microarray

**Figure 4:**
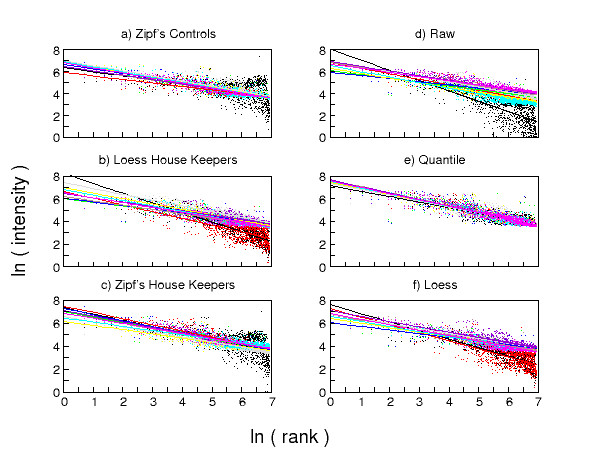
**Boutique microarray log plots. **Five mouse apoptosis boutique microarrays used in the mouse cell line experiments. Upper left to lower right: Log_e _median gene intensity vs. log_e _rank – **a**. Normalized according to Zipf's law, using internal positive and negative controls as proxies for the whole data set. **b**. Normalized with a loess curve fit using a selected set of housekeeping genes as proxies (see Methods). **c**. Normalized according to Zipf's law, using the same selected set of housekeeping genes as in b. as proxies **d**. The raw data. **e**. For comparison purposes only, normalized using the quantile method. **f**. For comparison purposes only, normalized using the standard loess method.

Plots of log_e _intensity versus log_e _rank fitted with linear regressions show that the Zipf's law normalization based on internal controls (Figure 4a) and on selected housekeeping genes (Figure 4c) have relatively similar coefficients *c *according to Zipf's power function (formula 1) as evidenced by the similarity in slopes of the regression lines. Loess normalization using selected housekeeping genes (Figure 4b) showed slightly more variation in *c *coefficients. The unnormalized raw data is also depicted (Figure 4d) along with two other normalization results, the loess method (Figure 4e) and the quantile method (Figure 4f). These are provided for reference only. Neither method can be validly applied to boutique arrays because both rely on the 'well-behaved' genes assumption.

It should be noted that much of the variation in *c *coefficients under the various normalization regimes is due to one channel (Cy3) on one microarray which had low median intensity and high variance due to low labelling efficiency (depicted in black in Figure 4). When normalized with the loess techniques (Figure 4c and 4f) the second channel (Cy5) on this array is adjusted to have a similar median intensity and variance, possibly skewing the results in favour of the Zipf's normalization techniques. To make the normalization method comparison unbiased, we eliminated this array from the analysis [see Additional file 3]. The Zipf's normalization based on internal controls (a) showed the lowest variation in *c *coefficients, the methods based on selected housekeeping genes (b, c) performed approximately equally well. Here again, raw (d), quantile normalized (e), and loess normalized (f) plots are provided for reference only.

**Figure 5:**
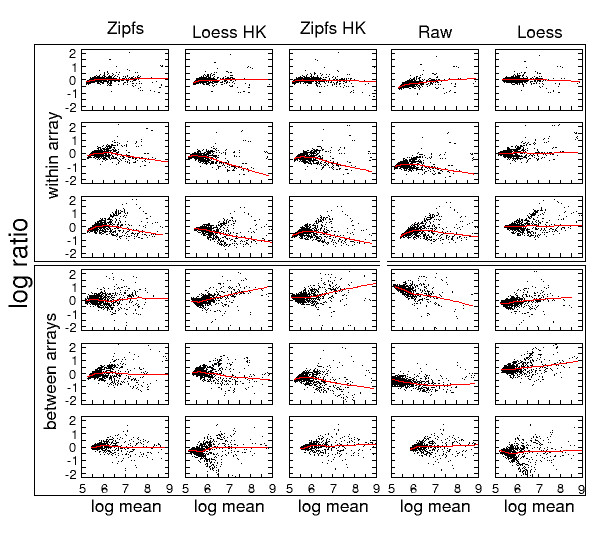
**Boutique microarray MA plots. **MA plots of the boutique data set comparing (in columns) Zipf's normalization using controls (Zipfs), Zipf's normalization using housekeepers (Zipfs HK), loess normalization using housekeepers (loess HK), raw data, and, for comparison purposes only, the standard loess normalization. Each row of plots represents one pairwise comparison, only 6 of the possible 45 pairwise comparisons of the 5 microarrays used in figure 4 are shown. The top three rows show within-array comparisons, and the bottom three rows show between-array comparisons. Lowess curves are plotted in red.

We generated MA plots for each of the normalization methods we compared (Figure 5). Typically, MA plots are produced from data from each channel of a single microarray. In addition to these 'within-array' plots (the first three rows of graphs in Figure 5), we also examined 'between-array' plots to evaluate the potential of the normalization methods to allow us to perform across array comparisons. The Zipf's using internal controls was slightly more well centered around the zero log ratio axis than the methods using selected housekeeping genes, especially in between-array plots. The raw and loess normalized plots are provided for reference only.

Finally, to quantify the differences between distributions after normalization, pairwise Kolmogorov-Smirnov values were computed for both the complete boutique array data set (Table 2c) and after eliminating the array which contained a low median intensity and high variance due to low labelling efficiency (Table 2d). In addition to computing the Kolmogorov-Smirnov values for all possible between-array pairwise combinations, we also summarized just the within-array pairwise comparisons (in parenthesis in Table 2). Of the normalization methods which can be applied to boutique microarrays, the Zipf's method using internal controls produced the most similar data distributions when all possible between-array comparisons are taken into consideration. When only within-array comparisons are considered, the Zipf's method using internal controls was better after the low labelling efficiency array was eliminated. The Zipf's method using selected housekeeping genes did not perform as well as the other methods in within-array comparisons, and was the middle performer when all possible between-array comparisons were computed. Kolmogorov-Smirnov values were computed from the global mean, Zipf's general, quantile, and loess normalization methods and are provided for reference only.

### Microarray platform comparison

**Figure 6:**
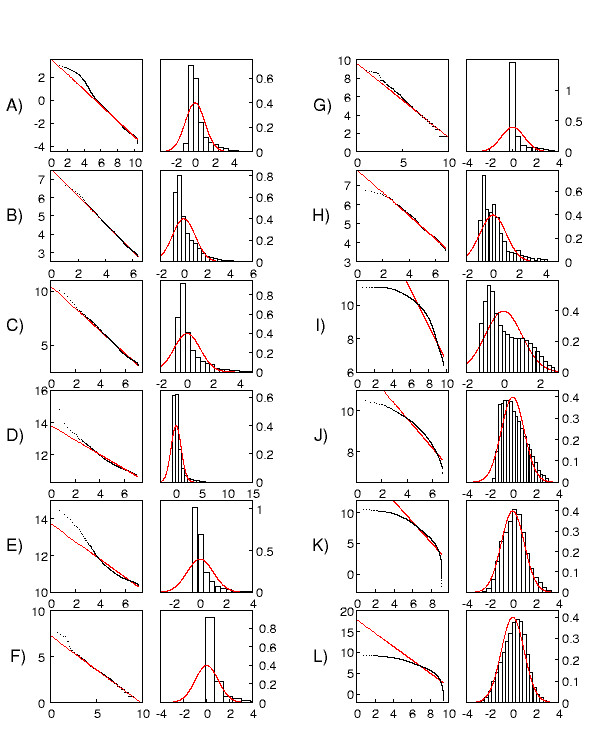
**Data set comparison. **Eleven microarray data sets (A-K) exhibiting varying degrees of conformation to power law and log normal distributions. On the left for each data set is a log mean intensity vs. log rank plot of the entire data set. Each array was sorted independently by intensity, and mean intensities for each rank over all arrays are plotted. A linear regression line is shown in red. Data sets with a linear distribution adhere well to a power law distribution. On the right for each data set is the distribution (ln(i) – μ) / σ of the mean intensities used in the left hand plots, where i is the mean measured intensity for each rank and μ and σ are the mean and variance of i respectively. The standard normal curve N(0,1) is shown in red for comparison. Data sets that display a standard normal distribution adhere well to a log normal distribution.

In a survey of seventeen microarray data sets, Hoyle *et. al*. [14] reported that microarray data follow a log-normal distribution with power-law tails. The three data sets presented in this paper exhibited distinct power-law distributions (Table 1, data sets A, B and H). To ascertain whether the data sets we used were unusual, we surveyed nine additional data sets (Table 1, data sets C-G, I-K) to determine their conformity to Zipf's law and the log-normal distribution respectively. The microarray data sets fell into two broad categories, power law distributions (Figure 6, data sets A-E) and log normal distributions (Figure 6, data sets I-K). Of the six power law data sets, two (B and C) clearly followed Zipf's law distributions. The remaining four (data sets A, D, E, and H), while still power-law distributed, showing noticeable deviations from the distribution at the lower rank (higher intensity) portion of the distribution. Of the platforms that where recognizably log normal in distribution, two fluorescent dye labeled, oligo-based Affymetrics platforms (data sets K and L) followed the distribution most closely and two dye labeled, cDNA systems (data sets I and J) were perceptibly log normal. The two SAGE experiments (data sets F and G) which were included for comparison purposes, exhibited Zipf's law distributions. Coefficients of determination (r^2^) of the log mean intensity vs. log rank are a measure of conformation to a power-law distribution and ranged from 0.9968 to 0.7773 for microarray data sets, 0.9982 and 0.9978 for the SAGE experiments (Table 1).

## Discussion

Zipf's law is based on observations made by linguist George Kingsley Zipf that the frequency of word occurrences in natural languages is proportional to the negative power of the rank order of the word. Beside the original findings in natural languages [15], Zipf's law has been found to apply to a plethora of natural phenomena, from the populations of cities to the impact factors of scientific journals as well as a variety of biological data, of which a review made available by Wentian Li [21] is an excellent online resource. It is important to point out, that being a phenomenological principle, Zipf's law does not imply that there is a universal underlying physical process at work. However, in much the same way that the Gaussian-Normal distribution occurs naturally in data and can be used to statistically test or otherwise manipulate the data, the fact that microarray data conforms to Zipf's law can be adapted for the purpose of microarray normalization.

Zipf's law is a power law function that states that the magnitude of an intensity measurement is inversely proportional to the rank of that data point in the data set, where c is a coefficient close to -1. Ranking is a method common in statistics, which has previously been used to analyze microarray data. Hoyle el. al. [14] used ranking as a method for evaluating microarray data and proposed the use of several statistics including χ^2 ^to quantify the agreement of the distribution to Benford's Law [22], and σ^2 ^as a quality control measure to detect such factors as low signal to background ratio, or mRNA probes extracted from mixed cell types. Ranking also figured prominently in the evaluation of a survey of inter-array normalization methods [23] where the statistics 'absolute rank deviation' and 'relative rank deviation' were used to select the method that produces the most 'well-normalized' data. The normalization procedure described in this paper is the first to combine these two ideas, namely that ranking can be used to judge the effectiveness of a normalization method, and that microarray data conforms to Zipf's law. We evolved these ideas into a novel and easily applicable normalization method and compared this method with existing methods to eliminate non-biological variation from microarray data sets.

In order to implement an appropriate data normalization technique, it is important to know the distribution of a given data set. Several publications have examined the data distributions that typically result from microarray experiments. In a survey of seventeen microarray data sets, sixteen of which were fluorescent dye labeled, Hoyle *et. al. *[14] reported that microarray data were found to have a log normal distributions with power law tails. More recent publications have reported that the abundance of expressed genes exhibit power-law distributions [11,13,24]. Results from our own data sets and a subsequent survey of publicly available data sets from both radioactively and fluorescently labeled platforms suggest that both types of distributions can be manifested in microarray data.

Comparisons between the Zipf's law and quantile normalization methods using MA plots showed that the quantile method effectively removes intensity dependant effects, sometimes referred to as 'banana' curves, from microarray data sets, while the Zipf's law method has no effect on the curved nature of the intensity dependent effect. This is not altogether unexpected as the quantile method was specifically designed to remove such effects. While the Zipf's method does not remove the curve from the intensity dependent effect, it does minimize negative consequences by rotating the data distribution such that the maximal number of data points lie on the zero of the log ratio axis. In this respect, the Zipf's law normalization technique can be considered inferior to the quantile method, however, it may still be a useful tool where the quantile method cannot be applied.

One such case, in which quantile normalization is inappropriate, is with so called 'boutique' microarrays where the genes spotted on the array represent a selected set of genes, for example from a specific pathway or those involved with a particular biological process or disease state. In such systems, most genes are expected to be differentially regulated when control and experimental samples are compared and the expected data distribution of control samples may be significantly different than that of experimental samples (in mean intensity for example). The quantile normalization method would effectively remove this difference by replacing the data distribution of each microarray with the mean distribution of all arrays. In contrast, the principle of normalization according to Zipf's law can also apply to arrays of this type if a group of control spots are included on the microarray. These control spots could be an external reference probe which hybridises to a concentration gradient of matching spots on the array, or internal positive (highly expressed genes) and negative (spotting buffer) control spots on the microarray, or an *a priori *selected set of housekeeping genes using a method such as that described by Wilson et. al. [4] or Schadt et. al. [25]. A linear model can be fitted to the control spots alone, and the normalization procedure can then be applied using the control spots as a proxy for the entire data distribution. The critical assumption in using control spots in normalization is establishing their relationship to the experimental spots.

The results of our comparison between methods which are designed to normalize boutique microarray data show that Zipf's law normalization using internal control spots results in a relatively well normalized data set when compared to Zipf's law normalization using selected housekeeping genes and the modified loess method using selected housekeeping genes. In addition, the Zipf's law method produced data distributions which are more similar between arrays allowing for between-array comparisons which are advantageous in terms of both cost, because of the reduced number of microarrays that need to be run, and, statistical power, by allowing for greater numbers (n), experimental design permitting.

## Conclusion

In summary, we examined the applicability of using Zipf's law as the basis for a novel normalization technique, which is applicable to both one channel microarray data and two channel microarrays. This method is shown to out-perform such methods as global normalization to the mean but would appear to be inferior to quantile normalization. The quantile method was superior to Zipf's law in removing intensity dependent effects commonly seen in microarray data. While the latter method cannot be applied to boutique arrays, we show that the Zipf's normalization method used with internal positive and negative controls or with selected housekeeping genes normalizes boutique arrays as well as currently existing methods. Additionally, data normalized with the Zipf's method using internal control spots seems more amenable to between-array gene intensity comparisons when compared to other methods.

## Methods

### Data acquisition

Data set A (Table 1) was generated using a global genome-wide cDNA clone set (Human UniGene clone set RZPD 1 Build 138, NCBI [26]), which consisted of ~33,792 cDNA clone inserts spotted in duplicate onto membranes [16]. These microarrays (n = 31) were hybridized with ^33^P-labeled cDNA derived from total RNA extracted from biopsy material from the sigmoidal colon of normal (control, n = 11), and patients with Crohn's disease (condition A, n = 10) and ulcerative colitis (condition B, n = 10). To emphasize that our normalization technique can be used to normalize other array systems, the second array set used was a smaller, but widely used, commercially available microarray system. Data set B (Table 1) was generated by using Atlas Rat cDNA microarrays (Clontech, 588 genes) probed with rat brain tissue, from control (cerebellum n = 10, olive n = 10) and harmaline treated (cerebellum n = 10, olive n = 9) animals. A third microarray data set, data set H (Table 1) was included to demonstrate the normalization method on two channel fluorescent based (Cy3/Cy5) oligonucleotide systems. These custom produced boutique microarrays (n = 5) contained 1024 spots, and were used in a study to identify differences in apoptotic mechanisms in two different mouse cell lines. Microarrays were probed according to established protocols and exposed to imaging plates overnight (BAS-MS 2325) and scanned at a 50 μm resolution on a FLA-3000G phosphoimager (Raytest, Germany). Image gridding was carried out using VisualGrid^® ^software [27], and intensity data was stored in a relational database and normalized and analyzed using database stored procedures and Perl scripts. All data was normalized from raw data, no background subtraction or other inter-array normalization was performed. Plots were generated using the Grace software package [28].

### Normalization

Normalization was accomplished by transforming the data such that the coefficient *c *and proportionality of the Zipf's power function (formula 1) are identical for all microarrays. This is easily achieved using a regression model on the log_e _intensity versus log_e _rank transformed data, which has the general form,

ln (*y*) = *a *+ *b*ln (*r*) + *e *    (2)

where *y *is the intensity, *r *is the rank, *a *is the regression constant (corresponding to proportionality in Zipf's power function), *b *is the regression coefficient (corresponding to the coefficient *c *in Zipf's power function), and *e *is an error coefficient, which is assumed to be normally distributed.

The first step in this three step procedure was to compute the median intensity of each gene over all microarrays to establish ranks, which were used as the 'reference' to which all microarrays were normalized. This was done by taking the median intensity (*y*_*med*_) of each gene, over all microarrays on which it was measured, and sorting the resulting list of medians to obtain their median ranks (*r*_*med*_). The regression model (2) is applied to the log_e _median intensities and their ranks to estimate *a*_*med *_and *b*_*med *_using the least squares method,



The ranking of genes by their median intensities effectively groups genes of similar overall expression level along the log rank axis. Under the assumptions that most genes are not differentially expressed, the reference curve generated from the median intensities should have an identical regression coefficient and constant to that of each individual microarray plotted using the ranks determined by the medians. For the genes which are differentially expressed, the median value represents a 'center' around which expression levels on each individual array may vary, and the neighbouring (by rank) genes, which do not (or only slightly) vary, act to stabilize the regression line and allow normalization to be performed.

In the second step of the normalization procedure, the regression model was applied individually to each microarray using the same ranking as the reference curve,



This results in a set of coefficients *a*_*k *_and *b*_*k *_which are estimated individually for each array using the least squares method, where k is equal to the number of microarrays in one channel systems, and equal to 2 time the number of microarrays (one for each channel) in two channel systems. Data from two channel arrays were treated in the same way as one channel systems, i.e. each channel was treated independently.

In the third step, the difference between the expected gene intensity value on the *k*th array and that of the reference curve was applied as the normalization factor,



A scaling factor was applied to the raw data before normalization such that the values *y*_*k*_,  and  were always greater than one to avoid negative values after log transformation. After normalization, the same scaling factor was applied to the data to back transform to their original magnitude. For example, if the smallest raw value in the data set was 0.1, the unlogged raw data was multiplied by a scaling factor of 10 before normalization, and the unlogged normalized data was divided by the same scaling after normalization.

In the special case of our third microarray data set (see Methods: Data Acquisition) which was a boutique array, the same procedure as described above was applied with the following modifications. Each microarray contained 32 spots each of internal positive controls (GAPDH, glyceraldehyde-3-phosphate_dehydrogenase) and internal negative controls (spotting buffer). The medians of all gene intensities were computed (including internal positive and negative controls), and median ranks were assigned as described. However, only the medians of the 64 internal control spots were used to estimate a_med _and b_med_, and only the 64 internal control spots from each array were used to estimate a_k _and b_k_. In both cases, the ranks generated from the entire data set, were used. The normalization factor was then applied over the entire data set as described above.

An alternative to the used of internal control spots for the normalization of boutique microarrays was also explored. Wilson, et. al. [4] described a method wherein a set of 'housekeeping' genes is selected *a priori *from the data set by virtue of their low variance in intensity and such that the entire range of intensities observed on the microarrays is uniformly represented. We also applied the Zipf's law normalization technique to our boutique microarrays using the set of housekeeping genes selected using the method of Wilson, et. al.

In addition to the normalization method based on Zipf's law, all data sets were normalized to a global mean (the mean of logged intensities from all microarrays) and the quantile method. The quantile method is applied by ranking the genes in each array by intensity, taking the median intensity at each rank, and replacing each gene intensity with the median intensity corresponding to the same rank. All normalization methods were compared to each other and to the raw data distribution using box plots and MA plots (pairwise array comparisons of the log-intensity ratio (M) to the mean log-intensity (A)). The two channel boutique microarray data set allowed further normalization methods not possible on one channel array systems to be applied. We normalized this data set using the popular loess method [19], and a modified Loess method specifically designed for boutique arrays using selected housekeeping genes described by Wilson, et. al. [4].

### Software

The Zipf's normalization procedure was initially implemented as an SQL stored procedure in a relational database. However, because this is not easily transferable to other systems, we provide two further implementations, a Perl script and an Excel macro [see Additional files 4, 5]. Implementations are available for download from our website [29] and as additional files accompanying this paper. Both the Perl script and Excel macro implement matrix algebra style computation, using either built-in functions or the Perl PDL module [30]. Normalization of two channel arrays with the loess method was performed using the marray package from R's Bioconductor [4]. Loess normalization using selected housekeeping genes and the selection of the housekeeping genes themselves was done with the tRMA package [19] which is publicly available for download on the internet. Sample data sets are also provided with this paper [see Additional files 6, 7, 8].

### Normalization method comparison

To compare and evaluate the effectiveness of the various normalization methods applied in this paper, several well established methods were used along with some less common techniques. MA plots [19] are a convenient way to examine differences in fluorescent marker efficiency and other dye effects in two channel microarray systems. In addition to the standard practice of generating within-array MA plots, we apply them additionally to one channel systems and between arrays in two channel systems to evaluate the extent to which a normalization procedure allows for multiple pairwise comparisons between microarrays. Plots of log_e _intensity versus log_e _rank fitted with linear regressions are a way to visually evaluate the normalization procedure according to the criteria of the Zipf's Law normalization. Specifically, all arrays have identical coefficients *c *and proportionality for the Zipf's power function when the slops and y-intercepts of the regression lines are identical. Finally, to quantify the similarity between microarray distributions after normalization, the mean Kolmogorov-Smirnov value was calculated over all possible pairwise combinations of microarrays within an experiment. In the case of two channel arrays, the mean of within-array Kolmogorov-Smirnov values was also computed (n = the number of arrays). It should be emphasized that even though the Kolmogorov-Smirnov values are technically a test statistic, no statistical test is performed. The values are here used only as a measure of similarity between microarray distributions.

### Microarray platform comparison

The underlying premise of the Zipf's normalization method is that microarray data distributions follow a power law distribution such that the relationship between the log intensities and the log ranks is clearly linear. While this assumption holds true for the three data sets we present in this paper, to evaluate the general applicability of the method we also examined eight publicly available data sets (Table 1, data sets C-G, I, K-L) from the NCBI Gene Expression Omnibus [18], and one unpublished data set from an independently maintained website [31] (Table 1, data set J). The survey contains a variety of microarray system types (cDNA vs. Oligo based, radioactivity vs. dye labeled systems, academic vs. commercially produced) and two SAGE experiments for comparison. Two plots were generated for each data set to ascertain the conformity to the Zipf's power law distribution and the log normal distribution respectively. For each data set, a representative array was constructed by ranking the intensities within each array, and then mean over ranks were taken. To determine how well data sets follow the Zipf's power law distribution, log intensity vs. log rank plots were constructed and linear regressions were performed. Data distributions, which were very linear in form, closely follow the power law distribution. A second plot of the distribution of (log y – μ) / σ, where y is the mean intensity over ranks, and μ and σ^2 ^are the mean and variance, was made for each data set to visualize the conformity to log normal distribution.

## List of abbreviations

EST – Expressed Sequence Tag

MA – log ratio (M) vs. mean log intensity (A)

NCBI – National Center for Biotechnology Information

RZPD – Deutsches Ressourcenzentrum für Genomforschung GmbH

SAGE – Serial analysis of gene expression

SQL – Structured Query Language

## Authors' contributions

TL conducted the data analysis and implementation of algorithms, participated in the development of the normalization method and is principle author of this manuscript. CMC generated the Unigene and Clontech microarray data set, participated in the development of the normalization method and participated in manuscript preparation. PJPC conceived of and participated in the development of the normalization method. RH participated in the generation of microarray data sets and participated in the development of the normalization method. GD conceived of and coordinated neurology related aspects of this study. SS conceived of and coordinated gastrointestinal related aspects of this study.

## Supplementary Material

Additional File 1 **Clontech microarray log plots **Five rat Clontech microarrays from the panel of thirty-nine microarrays probed with rat-brain tissue. Upper left to lower right: **a**. Log_e _median gene intensity vs. log_e _rank – conformity to Zipf's law is demonstrated by the linear regression line (in red) **b**. Five microarrays chosen to maximize pre-normalization variability, each plotted according to the gene ranks determined by their median gene intensity levels. **c**. The same five microarrays, normalized to a global median, with regression lines. **d**. The same five microarrays, normalized with the quantile method, with regression lines. **e**. The same five microarrays normalized taking Zipf's law into account, with regression lines. For plots b-d, a sub-sample of 50% of the data points are plotted for readability. Click here for file

Additional File 2 **Mean of squared log ratios from MA plots in **Figure 2 In Figure 2, it is difficult to see that the distribution of the Zipf's normalized data is more closely centered around zero on the log ratio axis than the Globally normalized data. To quantify this, the mean of squared log ratios was computed for each MA plot. The positions of the values in this table correspond exactly to the positions of the plots in Figure 2. In 6 out of 8 cases, the mean of squared log ratio is smaller in the Zipf's normalized data than in the corresponding Globally normalized data. Click here for file

Additional File 3 **Boutique microarray log plots **Four mouse apoptosis boutique microarrays used in the mouse cell line experiments. This is the same data set as shown in Figure 4, with the array containing one channel with low expression intensities and high variability removed. Upper left to lower right: Log_e _median gene intensity vs. log_e _rank – **a**. Normalized according to Zipf's law, using internal positive and negative controls as proxies for the whole data set. **b**. Normalized with a loess curve fit using a selected set of housekeeping genes as proxies (see Methods). **c**. Normalized according to Zipf's law, using the same selected set of housekeeping genes as in b. as proxies **d**. The raw data. **e**. For comparison purposes only, normalized using the quantile method. **f**. For comparison purposes only, normalized using the standard loess method. Click here for file

Additional File 4 Requires: Microsoft Excel (Does not handle missing data values.) Click here for file

Additional File 5 Requires: Perl (which runs on many platforms), the PDL perl module (Handles missing data values if PDL is compiled correctly.) Click here for file

Additional File 6 Microarray type: Filter based cDNA from the RZPD Number of genes: 33,792 Number of microarrays: 31 Probed with: Total RNA from human sigmoidal colon. Within microarray normalization: None Click here for file

Additional File 7 Microarray type: Clonetech Atlas Rat cDNA 7738-1 Number of genes: 558 Number of microarrays: 33 Probed with: Total RND from rat cerebellum and olive. Within microarray normalization: None Click here for file

Additional File 8 Microarray type: custom made glass slide Number of genes: 1024 Number of microarrays: 5 Probed with: Total RND from mouse cell lines. Within microarray normalization: None Click here for file

## Figures and Tables

**Table 1:** Data set comparison

**Set**	**Microarray Platform**	**Number of Data Points**	**Number of Expts**	**R2**	**GEO platform**	**GEO experiment**	**Array type**
A.	Human Unigene RZPD 1	34560	31	0.9877	GLP284	GSE1510	cDNA, membrane
B.	Clontech Atlas Rat cDNA Expression	588	39	0.9968	GPL158	GSE1509	cDNA, membrane
C.	Clontech Atlas Human 1.2 (I & II)	1176	10	0.9903	GPL127, GPL128	GSE751	cDNA, membrane
D.	Clontech Atlas Mouse 1.2	1159	12	0.9460	GPL144	GSE565	cDNA, membrane
E.	Clontech Atlas Human Cancer 1.2	1160	36	0.9109	GPL158	GSE796	cDNA, membrane
F.	NlaIII: Rattus norvegicus	76790	1	0.9982	GPL23	GSM1679	SAGE
G.	NlaIII: Homo sapiens	101677	1	0.9978	GPL4	GSM14771	SAGE
H.	Mouse Apoptosis	1024	5 × 2	0.994	--	--	cDNA, glass
I.	Caltech 16K cDNA mouse	908	58	0.8892	na	na	cDNA, glass
J.	Stanford Human Unigene	908	24	0.9081	na	na	cDNA, glass
K.	Affymetrix GeneChip Rat Genome	8799	24	0.8538	GPL85	GSE776	Oligo, glass
L.	Affymetrix GeneChip Human Genome	12625	24	0.7773	GPL91	GSE803	Oligo, glass

Eleven microarray data set comparison. Raw intensities, without background subtraction, were used. Controls and blanks were excluded. For Affymetrix chips (K and L), MM/PM ratios were used. For data set B two different Atlas arrays were analyzed together, when analyzed separately they gave similar results. For two channel array systems (I and J), each channel was treated as a separate array. For set I, only the cyanine-3 channel (spleen sample control) was used and for set J, both channels were used for analysis. Reference for data set J: Ross et. al. [31].

**Table 2:** Kolmogorov-Smirnov values

	**Microarray Platform**	**Pairwise Combinations (within array)**	**Raw**	**Global Median**	**Zipfs**	**Quantile**	**Loess**	**Loess HK**	**Zipfs Control**	**Zipfs HK**
a.	Clontech Atlas Rat cDNA Expression	465	0.539	0.484	0.119	0	na	na	na	na
b.	Human Unigene RZPD 1	703	0.662	0.225	0.060	0	na	na	na	na
c.	Mouse Apoptosis	45 (5)	0.548 (0.631)	**0.340 (0.318)**	**0.149 (0.167)**	**0 (0)**	**0.471 (0.042)**	0.487 (0.172)	0.182 (0.179)	0.303 (0.296)
d.	Mouse Apoptosis Subset	28 (4)	0.568 (0.667)	**0.303 (0.287)**	**0.111 (0.129)**	**0 (0)**	**0.317 (0.038)**	0.341 (0.190)	0.145 (0.128)	0.315 (0.291)

Three microarray data sets presented in this paper and seven normalization techniques were compared by computing the mean Kolmogorov-Smirnov values of all possible pairwise combinations of arrays within a data set. In the case of the two channel mouse apoptosis microarray, within-array pairwise comparisons were also computed and are shown in parenthesis (here n = the number of arrays, as each array has 2 channels). The symbol 'na' indicates that the normalization techniques which can only be carried out on two channel (loess) or boutique (loess HK, Zipfs Control, Zipfs HK) arrays were not performed on single channel arrays. Values in bold typeface were computed for reference purposes only – these normalization methods cannot be validly applied to boutique microarrays.
