# Supplementary figures and images for: Can Zipf's law be adapted to normalize microarrays?
Source: BMC Bioinformatics. 2005 Feb 23;6:37. doi: 10.1186/1471-2105-6-37 (PMC555536; doi:10.1186/1471-2105-6-37)

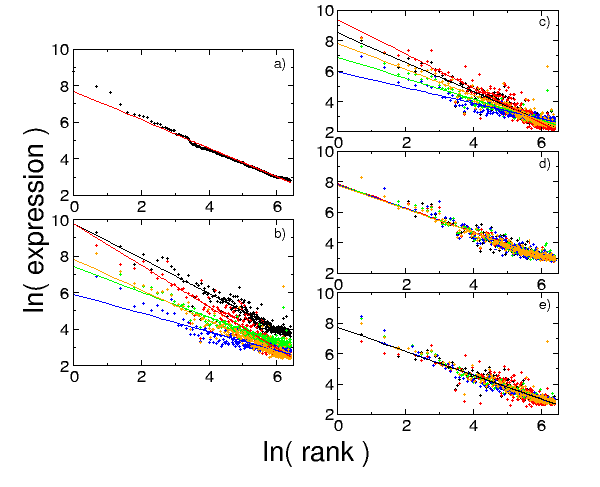

Supplement: Additional File 1 — Clontech microarray log plots Five rat Clontech microarrays from the panel of thirty-nine microarrays probed with rat-brain tissue. Upper left to lower right: a. Loge median gene intensity vs. loge rank – conformity to Zipf's law is demonstrated by the linear regression line (in red) b. Five microarrays chosen to maximize pre-normalization variability, each plotted according to the gene ranks determined by their median gene intensity levels. c. The same five microarrays, normalized to a global median, with regression lines. d. The same five microarrays, normalized with the quantile method, with regression lines. e. The same five microarrays normalized taking Zipf's law into account, with regression lines. For plots b-d, a sub-sample of 50% of the data points are plotted for readability. [file 1471-2105-6-37-S1.png]

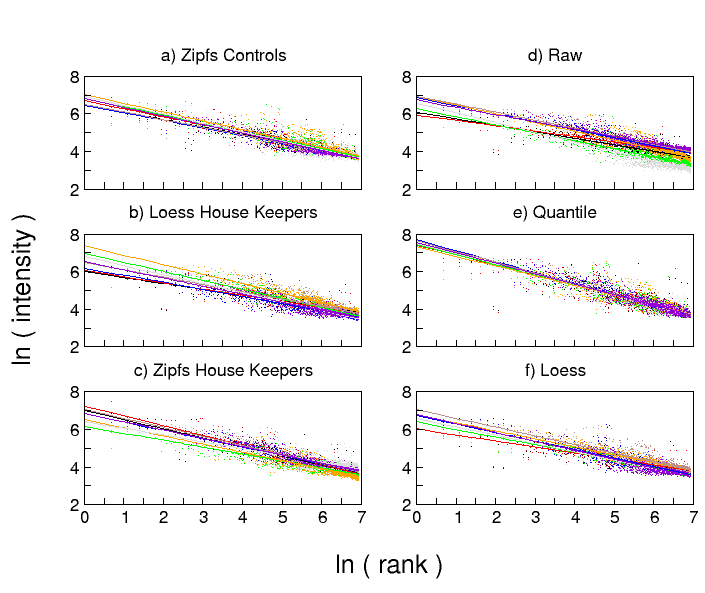

Supplement: Additional File 3 — Boutique microarray log plots Four mouse apoptosis boutique microarrays used in the mouse cell line experiments. This is the same data set as shown in Figure 4, with the array containing one channel with low expression intensities and high variability removed. Upper left to lower right: Loge median gene intensity vs. loge rank – a. Normalized according to Zipf's law, using internal positive and negative controls as proxies for the whole data set. b. Normalized with a loess curve fit using a selected set of housekeeping genes as proxies (see Methods). c. Normalized according to Zipf's law, using the same selected set of housekeeping genes as in b. as proxies d. The raw data. e. For comparison purposes only, normalized using the quantile method. f. For comparison purposes only, normalized using the standard loess method. [file 1471-2105-6-37-S3.png]
