# Supplementary material for: Can Zipf's law be adapted to normalize microarrays?
Source: BMC Bioinformatics. 2005 Feb 23;6:37. doi: 10.1186/1471-2105-6-37 (PMC555536; doi:10.1186/1471-2105-6-37)
Supplement: Additional File 2 — Mean of squared log ratios from MA plots in Figure 2 In Figure 2, it is difficult to see that the distribution of the Zipf's normalized data is more closely centered around zero on the log ratio axis than the Globally normalized data. To quantify this, the mean of squared log ratios was computed for each MA plot. The positions of the values in this table correspond exactly to the positions of the plots in Figure 2. In 6 out of 8 cases, the mean of squared log ratio is smaller in the Zipf's normalized data than in the corresponding Globally normalized data. [file 1471-2105-6-37-S2.doc]

**Supplemental Table 1 - Mean of squared log ratios from MA plots in Figure 2**

In Figure 2, it is difficult to see that the distribution of the Zipf’s normalized data is more closely centered around zero on the log ratio axis than the Globally normalized data. To quantify this, the mean of squared log ratios was computed for each MA plot. The positions of the values in this table correspond exactly to the positions of the plots in Figure 2. In 6 out of 8 cases, the mean of squared log ratio is smaller in the Zipf’s normalized data than in the corresponding Globally normalized data.

|  | Raw | Global | Zipf’s | Quantile |
| --- | --- | --- | --- | --- |
| MA plot 1 | 11.587 | 0.420 | 0.497 | 0.557 |
| MA plot 2 | 0.523 | 0.628 | 0.277 | 0.212 |
| MA plot 3 | 0.292 | 0.114 | 0.106 | 0.170 |
| MA plot 4 | 3.629 | 0.584 | 0.225 | 0.142 |
| MA plot 5 | 8.940 | 0.420 | 0.543 | 0.634 |
| MA plot 6 | 1.140 | 0.759 | 0.371 | 0.286 |
| MA plot 7 | 1.838 | 1.077 | 0.501 | 0.492 |
| MA plot 8 | 7.102 | 0.274 | 0.261 | 0.175 |
